# Supplementary material for: Surfactant amplifies yield-stress effects in the capillary instability of a film coating a tube
Source: J Fluid Mech. Author manuscript; Available in PMC 2023 Oct 5. (PMC7615153; doi:10.1017/jfm.2023.588)
Supplement: Appendices [file EMS181324-supplement-Appendices.pdf]

Data availability statement. The code used to generate the data in this study is openly available at https://doi.org/10.5281/zenodo.7794532.

Author ORCID. J.D. Shemilt, https://orcid.org/0000-0002-9158-0930; A. Horsley, https://orcid.org/0000-0003-1828-0058; O.E. Jensen, https://orcid.org/ 0000-0003-0172-6578; A.B. Thompson, https://orcid.org/ 0000-0001-9558-1554; C.A. Whitfield, https://orcid.org/ 0000-0001-5913-735X

Appendix A. Derivation of long-wave evolution equations

To derive evolution equations in the long-wave limit, we insert the scaled variables (2.22) into the dimensionless governing equations (2.10)-(2.21) and then truncate at leading order in δ. Mass and momentum conservation, to leading order, are

$$0 = \partial_{\bar{z}}\bar{w} + \frac{1}{r}\partial_r(r\bar{u}), \quad 0 = \partial_r p, \quad \partial_{\bar{z}} p = \frac{1}{r}\partial_r(r\bar{\tau}_{rz}). \qquad (A\,1a-c)$$

The no slip boundary conditions at the wall are

$$\bar{u} = \bar{w} = 0 \quad \text{on} \quad r = 1. \qquad (A\,2)$$

and the interfacial boundary conditions are

$$\partial_{\bar{t}}R + \bar{w}\partial_{\bar{z}}R = \bar{u}, \quad p = -\kappa[1 + \mathcal{M}(1-\Gamma)], \quad \bar{\tau}_{rz} = \mathcal{M}\partial_{\bar{z}}\Gamma \quad \text{on} \quad r = R. \qquad (A\,3a-c)$$

As discussed in §2.3, in (A 3b) we retain the full expression for κ (2.16) rather than truncating it. The surfactant transport equation (2.21) at leading order is

$$\partial_{\bar{t}}\,(R\Gamma) + \partial_{\bar{z}}\,(\bar{w}_s R\Gamma) = 0, \qquad (A\,4)$$

where $\bar{w}_s$ is the leading-order surface velocity. Using (A 1a), (A 2) and (A 3a), we can derive the evolution equation,

$$\partial_{\bar{t}}R = \frac{1}{R}\partial_{\bar{z}}\bar{Q}, \quad \text{where} \quad \bar{Q} = \int_R^1 \bar{w}r\,\mathrm{d}r. \qquad (A\,5)$$

The equations (A 4) and (A 5) are the long-wave evolution equations for R and Γ, but they need to be closed by deriving expressions for the surface velocity, $\bar{w}_s$, and the axial volume flux, $\bar{Q}$, which we pursue below. When presenting the equations (A 4) and (A 5) in §2.3, we rewrite them in terms of the unscaled variables (2.9), but they are entirely equivalent to the versions given here in terms of the scaled variables.

From (A 1b) and (A 3b), we deduce that

$$p(\bar{z},\bar{t}) = -\kappa\left[1 + \mathcal{M}(1-\Gamma)\right]. \qquad (A\,6)$$

Integrating (A 1c) and using (A 3c), we get an expression for the leading-order shear stress,

$$\bar{\tau}_{rz} = \frac{\partial_{\bar{z}}p}{2}\left(r - \frac{R^2}{r}\right) + \frac{R}{r}\mathcal{M}\partial_{\bar{z}}\Gamma. \qquad (A\,7)$$

Note that (A 7) holds independently of any rheological considerations. The non-zero components of the strain-rate tensor, up to $O(\delta^2)$, are

$$\dot{\gamma}_{rz} \sim \delta\partial_r\bar{w}, \quad \dot{\gamma}_{zz} \sim 2\delta^2\partial_{\bar{z}}\bar{w}, \quad \dot{\gamma}_{rr} \sim 2\delta^2\partial_r\bar{u}, \quad \dot{\gamma}_{\theta\theta} \sim \delta^2\frac{\bar{u}}{r}. \qquad (A\,8a-d)$$

Therefore, the constitutive relation (2.17) implies that, if $|\bar{\tau}_{rz}| > \bar{\mathcal{B}}$, where $\bar{\mathcal{B}} = \mathcal{B}/\delta$, then

$$\bar{\tau}_{rz} = \left(1 + \frac{\bar{\mathcal{B}}}{|\partial_r\bar{w}|}\right)\partial_r\bar{w}, \qquad (A\,9)$$

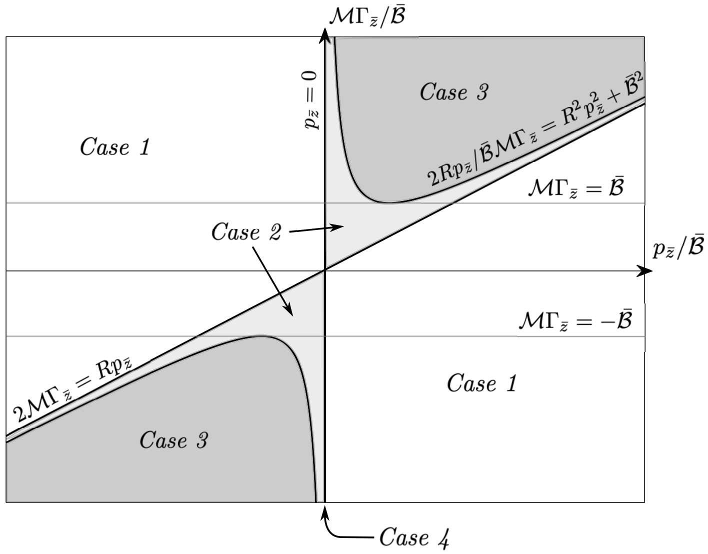

Figure 11: Map of $(p_{\bar z}/\bar{\mathcal{B}}, \mathcal{M}\Gamma_{\bar z}/\bar{\mathcal{B}})$-space with the shading indicating the locations of Cases 1-4, which are considered separately to derive the evolution equations in Appendix A. In this figure, subscripts denote derivatives. The map shown here, corresponds exactly with figure 2(b), which shows the location of the yielding types I-V. Unlike in figure 2(b), here the map is plotted in terms of scaled variables (2.22), but this just corresponds to a uniform scaling of the whole space by δ.

and the leading-order normal stresses are all of size $O(\delta)$. We call regions of the flow where $|\bar\tau_{rz}| > \bar{\mathcal{B}}$ shear-dominated, or fully-yielded. As identified by Balmforth & Craster (1999) for thin-film flows, regions where $|\bar\tau_{rz}| \leqslant \bar{\mathcal{B}}$ exhibit plug-like flow, with $\partial_r\bar w = 0$ to leading order. These plug-like regions can be yielded, with the normal stresses becoming $O(1)$ and the second invariant of the stress becoming exactly equal to $\bar{\mathcal{B}}$ to leading order. To derive an expression for $\bar w$ that holds everywhere in the layer, we will use (A 7) to determine the boundaries between the shear-dominated and plug-like regions, which are the surfaces satisfying $|\bar\tau_{rz}| = \bar{\mathcal{B}}$, then use (A 9) to find $\bar w$ in the shear-dominated regions and, finally, match these to the r-independent expression for $\bar w$ in the plug-like regions. Note that since $\bar\tau_{rz}$ is quadratic in r (A 7), there can be at most two shear-dominated regions and one plug-like region within $R \leqslant r \leqslant 1$. We will define the boundaries between shear-dominated and plug-like regions as $r = \Psi_\pm$, such that the shear-dominated regions are $R \leqslant r \leqslant \Psi_-$ and $\Psi_+ \leqslant r \leqslant 1$ and the plug-like region is $\Psi_- \leqslant r \leqslant \Psi_+$. Any of these three regions may not exist at a given point, in which case the width of it will be zero. There are four separate cases that we must consider separately below, depending on how many roots there are to each of the equations $\bar\tau_{rz} = \pm\bar{\mathcal{B}}$. Figure 11 illustrates where in $(p_{\bar z}/\bar{\mathcal{B}}, \mathcal{M}\Gamma_{\bar z}/\bar{\mathcal{B}})$-space each of these four cases occurs.

Case 1: $2\mathcal{M}\partial_{\bar z}\Gamma/(R\partial_{\bar z}p) < 1$ and $\partial_{\bar z}p \neq 0$. Figure 11 shows that this case occurs in the upper-left and lower-right regions of $(p_{\bar z}/\bar{\mathcal{B}}, \mathcal{M}\Gamma_{\bar z}/\bar{\mathcal{B}})$-space, where capillary and Marangoni forces either act in opposite directions or, if they act in the same direction, the Marangoni force is relatively weak.

In this case, $\bar\tau_{rz}$ is monotonic for $r > 0$, so there is exactly one solution to $\bar\tau_{rz} = \bar{\mathcal{B}}$ and exactly one solution to $\bar\tau_{rz} = -\bar{\mathcal{B}}$. We define $r = \psi_\pm^{(1)}$ as the surfaces such that $\bar\tau_{rz} =$

$\pm\bar{\mathcal{B}}\,\mathrm{sgn}(\partial_{\bar{z}}p)$. From (A 7), we get

$$\psi_{\pm}^{(1)} = \pm\frac{\bar{\mathcal{B}}}{|\partial_{\bar{z}}p|} + \sqrt{\left(\frac{\bar{\mathcal{B}}}{\partial_{\bar{z}}p}\right)^2 + R^2 - \frac{2R\mathcal{M}\partial_{\bar{z}}\Gamma}{\partial_{\bar{z}}p}}. \tag{A 10}$$

The functions $\psi_{\pm}^{(1)}$ could take any positive value, and whether the surfaces $r=\psi_{\pm}^{(1)}$ lie within the layer, $R<r<1$, or not determines which of the shear-dominated and plug-like regions exist. If both surfaces lie within the layer, i.e. $R<\psi_{-}^{(1)}\leqslant\psi_{+}^{(1)}<1$, then $\Psi_{\pm}=\psi_{\pm}^{(1)}$ and both shear-dominated regions and the plug-like region exist. We say that the layer is exhibiting yielding of type I (see figure 2a). If, instead, $\psi_{-}^{(1)}\leqslant R<\psi_{+}^{(1)}<1$ then $\Psi_{+}=\psi_{+}^{(1)}$ and $\Psi_{-}=R$, and there is yielding of type II. If $\psi_{\pm}^{(1)}\leqslant R$ then $\Psi_{\pm}=R$ and there is yielding of type III. Similarly, if $\psi_{\pm}^{(1)}\geqslant 1$ then $\Psi_{\pm}=1$ and there is also yielding of type III. If $R<\psi_{-}^{(1)}<1\leqslant\psi_{+}^{(1)}$, then $\Psi_{-}=\psi_{-}^{(1)}$ and $\Psi_{+}=1$, and there is yielding of type IV. Finally, if $\psi_{-}^{(1)}<R<1<\psi_{+}^{(1)}$ then $\Psi_{-}=R$, $\Psi_{+}=1$ and there is no yielding (type V). We can identify that all of the above cases are captured by defining

$$\Psi_{\pm} = \max\left[R, \min\left(1, \psi_{\pm}^{(1)}\right)\right]. \tag{A 11}$$

With the expressions for $\Psi_{\pm}$ now established, we can proceed to determine $\bar{w}$. In $\Psi_{+}\leqslant r\leqslant 1$, we equate (A 7) with (A 9), solve for $\partial_r\bar{w}$, then integrate and use (A 2). We also need to use the fact that $\mathrm{sgn}(\partial_r\bar{w})=\mathrm{sgn}(\partial_{\bar{z}}p)$ in $\Psi_{+}\leqslant r\leqslant 1$. Doing this gives

$$\bar{w} = \frac{\partial_{\bar{z}}p}{4}\left(r^2-1-2R^2\log r\right) + \mathcal{M}(\partial_{\bar{z}}\Gamma)R\log r - \bar{\mathcal{B}}\,\mathrm{sgn}(\partial_{\bar{z}}p)(r-1) \tag{A 12}$$

for $\Psi_{+}\leqslant r\leqslant 1$. We then determine the velocity in the plug-like region, $\Psi_{-}\leqslant r\leqslant\Psi_{+}$, by evaluating (A 12) at $r=\Psi_{+}$. Therefore, $\bar{w}=\bar{w}_p(\bar{z},\bar{t})$ in $\Psi_{-}\leqslant r\leqslant\Psi_{+}$, where

$$\bar{w}_p = \frac{\partial_{\bar{z}}p}{4}\left(\Psi_{+}^2-1-2R^2\log\Psi_{+}\right) + \mathcal{M}(\partial_{\bar{z}}\Gamma)R\log\Psi_{+} - \bar{\mathcal{B}}\,\mathrm{sgn}(\partial_{\bar{z}}p)(\Psi_{+}-1). \tag{A 13}$$

From (A 13), we have the value of $\bar{w}$ at $r=\Psi_{-}$, which can be used to find $\bar{w}$ in the shear-dominated region, $R\leqslant r\leqslant\Psi_{-}$, using the same procedure as above, but noting that instead $\mathrm{sgn}(\partial_r\bar{w})=-\,\mathrm{sgn}(\partial_{\bar{z}}p)$. We get

$$\bar{w} = \bar{w}_p + \frac{\partial_{\bar{z}}p}{4}\left[r^2-\Psi_{-}^2-2R^2\log\left(\frac{r}{\Psi_{-}}\right)\right] + \mathcal{M}(\partial_{\bar{z}}\Gamma)R\log\left(\frac{r}{\Psi_{-}}\right) + \bar{\mathcal{B}}\,\mathrm{sgn}(\partial_{\bar{z}}p)(r-\Psi_{-}) \tag{A 14}$$

for $R\leqslant r\leqslant\Psi_{-}$. This then completes the leading-order axial velocity in Case 1. We can then integrate to determine volume flux, $\bar{Q}$, and evaluate (A 14) at $r=R$ to get the surface velocity, $\bar{w}_s$. This gives

$$\bar{Q} = -\frac{\partial_{\bar{z}}p}{16}F_1 - \frac{1}{4}\mathcal{M}(\partial_{\bar{z}}\Gamma)RF_2 - \frac{\bar{\mathcal{B}}}{6}\,\mathrm{sgn}(\partial_{\bar{z}}p)(F_3+F_4), \tag{A 15}$$

where the functions F_1, F_2, F_3 and F_4 are given in (2.29), and

$$\bar{w}_s = \frac{\partial_{\bar{z}}p}{4}G_1 + \mathcal{M}(\partial_{\bar{z}}\Gamma)RG_2 + \bar{\mathcal{B}}\,\mathrm{sgn}(\partial_{\bar{z}}p)(G_3+G_4), \tag{A 16}$$

where the functions G_1, G_2, G_3 and G_4 are given in (2.32).

Case 2: $1+\bar{\mathcal{B}}^2/(R\partial_{\bar{z}}p)^2\geqslant 2\mathcal{M}\partial_{\bar{z}}\Gamma/(R\partial_{\bar{z}}p)\geqslant 1$ and $\partial_{\bar{z}}p\neq 0$. Again, figure 11 illustrates the region of $(p_{\bar{z}}/\bar{\mathcal{B}}, \mathcal{M}\Gamma_{\bar{z}}/\bar{\mathcal{B}})$-space where this case occurs. In this case, there are no solutions to $\bar{\tau}_{rz}=-\bar{\mathcal{B}}\,\mathrm{sgn}(\partial_{\bar{z}}p)$ in $r\geqslant 0$. We define $r=\psi_{\pm}^{(2)}$ as the two surfaces on which

$\bar{\tau}_{rz} = \bar{\mathcal{B}}\,\mathrm{sgn}(\partial_{\bar{z}} p)$, with $\psi_-^{(2)} \leqslant \psi_+^{(2)}$. From (A 7), these are

$$\psi_\pm^{(2)} = \frac{\bar{\mathcal{B}}}{|\partial_{\bar{z}} p|} \pm \sqrt{\left(\frac{\bar{\mathcal{B}}}{\partial_{\bar{z}} p}\right)^2 + R^2 - \frac{2RM\partial_{\bar{z}}\Gamma}{\partial_{\bar{z}} p}}. \tag{A 17}$$

As in Case 1 above, by considering all possible types of yielding that can occur, we find

$$\Psi_\pm = \max\left[R, \min\left(1, \psi_\pm^{(2)}\right)\right]. \tag{A 18}$$

We can then proceed similarly to above to derive $\bar{w}$. The only difference here is that $\partial_r \bar{w}$ has the opposite sign in the near-interface shear-dominated region, $R \leqslant r \leqslant \Psi_-$, compared to Case 1. Hence, $\bar{w}$ is still given by (A 12) in $\Psi_+ \leqslant r \leqslant 1$, and by (A 13) in $\Psi_- \leqslant r \leqslant \Psi_+$. However, we have

$$\bar{w} = \bar{w}_p + \frac{\partial_{\bar{z}} p}{4}\left[r^2 - \Psi_-^2 - 2R^2 \log\left(\frac{r}{\Psi_-}\right)\right] + \mathcal{M}(\partial_{\bar{z}}\Gamma) R \log\left(\frac{r}{\Psi_-}\right) - \bar{\mathcal{B}}\,\mathrm{sgn}(\partial_{\bar{z}} p)(r - \Psi_-) \tag{A 19}$$

in $R \leqslant r \leqslant \Psi_-$. This leads to slightly modified expressions for axial volume flux,

$$\bar{Q} = -\frac{\partial_{\bar{z}} p}{16} F_1 - \frac{1}{4}\mathcal{M}(\partial_{\bar{z}}\Gamma) R F_2 - \frac{\bar{\mathcal{B}}}{6}\,\mathrm{sgn}(\partial_{\bar{z}} p)(F_3 - F_4), \tag{A 20}$$

and surface velocity,

$$\bar{w}_s = \frac{\partial_{\bar{z}} p}{4} G_1 + \mathcal{M}(\partial_{\bar{z}}\Gamma) R G_2 + \bar{\mathcal{B}}\,\mathrm{sgn}(\partial_{\bar{z}} p)(G_3 - G_4), \tag{A 21}$$

in Case 2.

Case 3: $2\mathcal{M}\partial_{\bar{z}}\Gamma/(R\partial_{\bar{z}} p) > 1 + \bar{\mathcal{B}}^2/(R\partial_{\bar{z}} p)^2$. Figure 11 shows that, in this case, capillary and Marangoni forces act in the same direction, with Marangoni forces being relatively strong. In Case 3, $|\bar{\tau}_{rz}| > \bar{\mathcal{B}}$ for all $r > 0$. Hence, the whole layer is shear-dominated (yield type III). We set $\Psi_+ = \Psi_- = R$. The axial velocity, $\bar{w}$ is then given by (A 12) for the whole layer, $R \leqslant r \leqslant 1$. Since there is no contribution from the plug-like region or the near-interface shear-dominated region, the expressions for $\bar{Q}$ and $\bar{w}_s$ from Case 1, (A 15) and (A 16), and from Case 2, (A 20) and (A 21), both recover the correct expressions for $\bar{Q}$ and $\bar{w}_s$ in Case 3, so either can be used.

Case 4: $\partial_{\bar{z}} p = 0$. When $\partial_{\bar{z}} p = 0$, there is exactly one solution to $\bar{\tau}_{rz} = \bar{\mathcal{B}}\,\mathrm{sgn}(\partial_{\bar{z}}\Gamma)$ and no other solutions to $|\bar{\tau}_{rz}| = \bar{\mathcal{B}}$ in $r > 0$. The solution corresponds to $r = \Psi_-$. We can deduce from (A 7) that $\Psi_- = \min(1, \max[R, R\mathcal{M}|\Gamma_z|/\bar{\mathcal{B}}])$, and we always have $\Psi_+ = 1$. This is consistent with the behaviour in Cases 1 and 2 as $\partial_{\bar{z}} p \to 0$. The velocity, $\bar{w}$, can be derived using a similar procedure as in the cases above: we equate (A 7) and (A 9) in $R \leqslant r \leqslant \Psi_-$, solve for $\partial_r \bar{w}$, then integrate and apply no slip at $r = \Psi_-$ to get $\bar{w}$. We have $\bar{w} = 0$ in $\Psi_- \leqslant r \leqslant 1$. Again, we integrate the velocity to get the flux,

$$\bar{Q} = -\frac{1}{4} R\mathcal{M}(\partial_{\bar{z}}\Gamma) F_2 + \bar{\mathcal{B}}\,\mathrm{sgn}(\partial_{\bar{z}}\Gamma) F_4, \tag{A 22}$$

and evaluate $\bar{w}$ at $r = R$ to get the surface velocity,

$$\bar{w}_s = \mathcal{M}(\partial_{\bar{z}}\Gamma) R G_2 - \bar{\mathcal{B}}\,\mathrm{sgn}(\partial_{\bar{z}}\Gamma) G_4, \tag{A 23}$$

in Case 4.

We have now derived expressions for the axial volume flux and surface velocity in all cases, and so have closed the evolution equations (A 4) and (A 5). Finally, the lateral boundary

conditions (2.8), at leading order in δ, imply

$$\partial_{\bar{z}} R = \bar{Q} = \bar{w}_s \Gamma \quad \text{on} \quad \bar{z} = \bar{L}. \tag{A 24}$$

Equations (2.27)-(2.33), are presented in terms of the unscaled variables (2.9), but they are entirely equivalent to what is derived here.

Appendix B. Rankine-Hugoniot condition for shock propagation speed

Suppose we observe a jump discontinuity in Γ_z at the point $z = z_s(t)$, and denote the size of the jump by $[\Gamma_z]_-^+$. Then we can use the following argument to derive a Rankine-Hugoniot condition (see, e.g., Billingham & King (2001)) for the speed of propagation of the discontinuity. Define two points, z_1 and z_2, such that $0 < z_1 < z_s(t) < z_2 < L$. Then the surfactant transport equation (2.43) implies

$$\frac{\mathrm{d}}{\mathrm{d}t} \int_{z_1}^{z_2} \Gamma_z \,\mathrm{d}z = -\left[(\tilde{w}_s \Gamma)_z\right]_{z_1}^{z_2}. \tag{B 1}$$

Splitting the integral in (B 1) and expanding, we get

$$\frac{\mathrm{d}}{\mathrm{d}t}\left(\int_{z_1}^{z_s} \Gamma_z \,\mathrm{d}z + \int_{z_s}^{z_2} \Gamma_z \,\mathrm{d}z\right) = -\frac{\mathrm{d}z_s}{\mathrm{d}t}\,[\Gamma_z]_{z_1}^{z_2} + \int_{z_1}^{z_s} \Gamma_{tz} \,\mathrm{d}z + \int_{z_s}^{z_2} \Gamma_{tz} \,\mathrm{d}z. \tag{B 2}$$

Then taking $z_1 \to z_s^-$ and $z_2 \to z_s^+$, (B 1) and (B 2) imply

$$\frac{\mathrm{d}z_s}{\mathrm{d}t}\,[\Gamma_z]_-^+ = \left[(\tilde{w}_s \Gamma)_z\right]_-^+, \tag{B 3}$$

which defines the shock propagation speed

$$u_s \equiv \frac{\mathrm{d}z_s}{\mathrm{d}t} = \frac{\left[(\tilde{w}_s \Gamma)_z\right]_-^+}{[\Gamma_z]_-^+}. \tag{B 4}$$

The velocity (B 4) can, in theory, be integrated to get the shock location,

$$z_s(t) = z_s(t_0) + \int_{t_0}^{t} u_s(t')\mathrm{d}t', \tag{B 5}$$

if the location of the shock is known at some time, t_0. We have used (B 5) to provide a consistency check on numerical simulations. For example, for the simulation shown in figure 3(a), we take $t_0 = 69.2$, which is shortly after the interface-adjacent yielded region first appears, we take the value of $z_s(t_0)$ from the simulation and we use the values for $[\tilde{w}_s \Gamma_z]_-^+$ and $[\Gamma_z]_-^+$ also from the simulation. Doing this, we find that the prediction of the location of either shock computed via (B 5) never deviates from the shock location in the simulation by more than three grid points (when using 200 points), providing evidence that the numerical scheme is capturing the speed of shock propagation. Since we can only calculate u_s by using values from the numerical simulation this does not provide independent validation of the numerical method. However, it does provide evidence that at each instant the shock propagation velocity is being accurately calculated in the numerical scheme from the finite differenced derivatives and that spurious behaviour is not being introduced by the numerical scheme.

Appendix C. Thin-film dynamics at large M

Consider the thin-film equations (2.41)-(2.45) in the limit of very strong surfactant. We propose the expansions

$$\left.\begin{array}{lll} H = \mathcal{H}_0 + \frac{1}{M}\mathcal{H}_1 + \ldots, & \tilde{p} = \tilde{p}_0 + \frac{1}{M}\tilde{p}_1 + \ldots, & \tilde{w}_s = \tilde{w}_0 + \frac{1}{M}\tilde{w}_1 + \ldots, \\ \Gamma = \tilde{\mathcal{G}}_0 + \frac{1}{M}\tilde{\mathcal{G}}_1 + \ldots, & Y_- = Y_{-0} + \frac{1}{M}Y_{-1} + \ldots, & Y_+ = Y_{+0} + \frac{1}{M}Y_{+1} + \ldots, \end{array}\right\} \tag{C 1}$$

as $M \to \infty$. The Marangoni force at the interface must be finite in the limit $M \to \infty$, so we require $|M\Gamma_z| < \infty$. This implies $\tilde{\mathcal{G}}_{0,z} = 0$ and conservation of total mass of surfactant then means we must have

$$\tilde{\mathcal{G}}_0 = 1. \tag{C 2}$$

Inserting (C 1) into the surfactant transport equation (2.43), and using (C 2), implies $\tilde{w}_{0,z} = 0$. Combined with the boundary condition (2.45) which enforces $\tilde{w}_0\tilde{\mathcal{G}}_0 = 0$ at $z = \{0, L\}$, we get

$$\tilde{w}_0 = 0. \tag{C 3}$$

This means that in the limit of strong surfactant, the interface of the thin film is essentially immobilised by Marangoni effects. Inserting (C 3) into the expression for surface velocity (2.44), and rearranging gives

$$\mathcal{H}_0 - Y_{+0} = Y_{-0}, \tag{C 4}$$

assuming $\tilde{p}_{0,z} \neq 0$. The result (C 4) says that the wall-adjacent and interface-adjacent fully yielded regions always have the same thickness in the leading-order theory. This also means that only yielding of types I or V can occur. We will assume for now that the fluid is yielded, so $Y_- > 0$ and $Y_+ < H$, and then subsequently present criteria for when the fluid rigidifies.

The definition (2.40) gives

$$Y_{\pm 0} = \mathcal{H}_0 \pm \frac{B}{|\tilde{p}_{0,z}|} + \frac{\tilde{\mathcal{G}}_{1,z}}{\tilde{p}_{0,z}}, \tag{C 5}$$

which when combined with (C 4), implies

$$\tilde{\mathcal{G}}_{1,z} + \frac{1}{2}\mathcal{H}_0\tilde{p}_{0,z} = 0. \tag{C 6}$$

Note that, for certain choices of initial conditions, $\tilde{\mathcal{G}}_1$ may not initially satisfy (C 6), in which case we expect there to be a rapid adjustment at early times to a state where (C 6) is satisfied. For an arbitrary choice of initial Γ, the change in Γ required to reach a state satisfying (C 6) is of size $O(1/M)$, so we expect the time scale for the adjustment to this state to be of size $O(1/M)$ also, since the initial surface velocity would be of size $O(1)$ during the adjustment period.

At leading order, the axial volume flux (2.42), is

$$q = \frac{1}{6}\tilde{p}_{0,z}Y_{-0}^2\left(2Y_{-0} - 3\mathcal{H}\right). \tag{C 7}$$

If we define the function

$$\tilde{\mathcal{Y}} \equiv \frac{1}{2}H - \frac{B}{|\tilde{p}_z|}, \tag{C 8}$$

then from (C 8), $Y_{-0} = \tilde{\mathcal{Y}}$ to leading order when the fluid is yielded. Note that, from (C 4), the criterion for no yielding to occur in the leading order theory is $Y_{-0} = 0$. Therefore, from (2.41) and (C 7), the leading order evolution equation, which holds if fluid is yielded or unyielded, is (3.9). To reach (3.9), we have assumed so far that $\tilde{p}_z \neq 0$, but we can see from

(C 6) that if $\tilde{p}_{0,z} = 0$ then $\tilde{\mathcal{G}}_{1,z} = 0$ so there is no driving force. Therefore, (3.9) also holds when $\tilde{p}_z = 0$ since it says there is no motion in that case.

REFERENCES

Ahmadikhamsi, S., Golfier, F., Oltean, C., Lefèvre, E. & Bahrani, S.A. 2020 Impact of surfactant addition on non-newtonian fluid behavior during viscous fingering in hele-shaw cell. *Phys. Fluids* **32** (1), 012103.

Balmforth, N.J. & Craster, R.V. 1999 A consistent thin-layer theory for Bingham plastics. *J. Non-Newton. Fluid Mech.* **84** (1), 65–81.

Balmforth, N.J., Ghadge, S. & Myers, T.G. 2007 Surface tension driven fingering of a viscoplastic film. *J. Non-Newton. Fluid Mech.* **142** (1), 143–149.

Billingham, J. & King, A.C. 2001 *Wave Motion*. Cambridge University Press.

Camassa, R., Forest, M.G., Lee, L., Ogrosky, H.R. & Olander, J. 2012 Ring waves as a mass transport mechanism in air-driven core-annular flows. *Phys. Rev. E* **86** (6), 066305.

Camassa, R. & Ogrosky, H.R. 2015 On viscous film flows coating the interior of a tube: thin-film and long-wave models. *J. Fluid Mech.* **772**, 569–599.

Camassa, R., Ogrosky, H.R. & Olander, J. 2014 Viscous film flow coating the interior of a vertical tube. Part 1. Gravity-driven flow. *J. Fluid Mech.* **745**, 682–715.

Camassa, R., Ogrosky, H.R. & Olander, J. 2017 Viscous film-flow coating the interior of a vertical tube. Part 2. Air-driven flow. *J. Fluid Mech.* **825**, 1056–1090.

Carroll, B.J. & Lucassen, J. 1974 Effect of surface dynamics on the process of droplet formation from supported and free liquid cylinders. *J. Chem. Soc., Faraday Trans. 1* **70**, 1228–1239.

Cassidy, K. J., Halpern, D., Ressler, B. G. & Grotberg, J. B. 1999 Surfactant effects in model airway closure experiments. *J. Appl. Physiol.* **87** (1), 415–427.

Chen, Z., Zhong, M., Luo, Y., Deng, L., Hu, Z. & Song, Y. 2019 Determination of rheology and surface tension of airway surface liquid: a review of clinical relevance and measurement techniques. *Respir. Res.* **20** (1), 274.

Craster, R.V. & Matar, O.K. 2000 Surfactant transport on mucus films. *J. Fluid Mech.* **425**, 235–258.

Erken, O., Fazla, B., Muradoglu, M., Izbassarov, D., Romanò, F. & Grotberg, J.B. 2023 Effects of elastoviscoplastic properties of mucus on airway closure in healthy and pathological conditions. *Phys. Rev. Fluids* **8**, 053102.

Everett, D.H. & Haynes, J.M. 1972 Model studies of capillary condensation. I. Cylindrical pore model with zero contact angle. *J. Colloid Interface Sci.* **38** (1), 125–137.

Gauglitz, P.A. & Radke, C.J. 1988 An extended evolution equation for liquid film breakup in cylindrical capillaries. *Chem. Eng. Sci.* **43** (7), 1457–1465.

Glasser, A., Cloutet, É., Hadziioannou, G. & Kellay, H. 2019 Tuning the rheology of conducting polymer inks for various deposition processes. *Chem. Mater.* **31** (17), 6936–6944.

Griese, M., Essl, R., Schmidt, R., Rietschel, E., Ratjen, F., Ballman, M. & Paul, K. 2004 Pulmonary surfactant, lung function, and endobronchial inflammation in cystic fibrosis. *Am. J. Resp. Crit. Care Med.* **170** (9), 1000–1005.

Gunasekara, L. et al. 2017 Pulmonary surfactant dysfunction in pediatric cystic fibrosis: mechanisms and reversal with a lipid-sequestering drug. *J. Cyst. Fibros.* **16** (5), 565–572.

Halpern, D. & Frenkel, A.L. 2003 Destabilization of a creeping flow by interfacial surfactant: linear theory extended to all wavenumbers. *J. Fluid Mech.* **485**, 191–220.

Halpern, D., Fujioka, H. & Grotberg, J.B. 2010 The effect of viscoelasticity on the stability of a pulmonary airway liquid layer. *Phys. Fluids* **22** (1), 011901.

Halpern, D. & Grotberg, J.B. 1992 Fluid-elastic instabilities of liquid-lined flexible tubes. *J. Fluid Mech.* **244**, 615–632.

Halpern, D. & Grotberg, J.B. 1993 Surfactant effects on fluid-elastic instabilities of liquid-lined flexible tubes: A model of airway closure. *J. Biomech Eng.* **115** (3), 271–277.

Halpern, D. & Grotberg, J.B. 2003 Nonlinear saturation of the Rayleigh instability due to oscillatory flow in a liquid-lined tube. *J. Fluid Mech.* **492**, 251–270.

Hammond, P.S. 1983 Nonlinear adjustment of a thin annular film of viscous fluid surrounding a thread of another within a circular cylindrical pipe. *J. Fluid Mech.* **137**, 363–384.

Hewitt, I.J. & Balmforth, N.J. 2012 Viscoplastic lubrication theory with application to bearings and the washboard instability of a planing plate. *J. Non-Newton. Fluid Mech.* **169-170**, 74–90.

Hill, D.B., Button, B., Rubinstein, M. & Boucher, R.C. 2022 Physiology and pathophysiology of human airway mucus. *Physiol. Rev.* **102** (4), 1757–1836.

Hohlfield, J.M. 2002 The role of surfactant in asthma. *Respir. Res.* **3** (1), 4.

Hsia, Connie C.W., Hyde, Dallas M. & Weibel, Ewald R. 2016 Lung structure and the intrinsic challenges of gas exchange. In *Comprehensive Physiology*, 1st edn. (ed. Ronald Terjung), pp. 827–895. Wiley.

Huh, D., Fujioka, H., Tung, Y., Futai, N., Paine, R., Grotberg, J.B. & Takayama, S. 2007 Acoustically detectable cellular-level lung injury induced by fluid mechanical stresses in microfluidic airway systems. *Proc. Natl. Acad. Sci. U.S.A.* **104** (48), 18886–18891.

Jalaal, M. 2016 Controlled spreading of complex droplets. PhD thesis, University of British Columbia.

Jalaal, M. & Balmforth, N.J. 2016 Long bubbles in tubes filled with viscoplastic fluid. *J. Non-Newton. Fluid Mech.* **238**, 100–106.

Jalaal, M., Stoeber, B. & Balmforth, N.J. 2021 Spreading of viscoplastic droplets. *J. Fluid Mech.* **914**, A21.

Johnson, M., Kamm, R.D., Ho, L.W., Shapiro, A. & Pedley, T.J. 1991 The nonlinear growth of surface-tension-driven instabilities of a thin annular film. *J. Fluid Mech.* **233**, 141–156.

van der Kolk, J., Tieman, D. & Jalaal, M. 2023 Viscoplastic lines: printing a single filament of yield stress material on a surface. *J. Fluid Mech.* **958**, A34.

Lai, S.K., Wang, Y., Wirtz, D. & Hanes, J. 2009 Micro- and macrorheology of mucus. *Adv. Drug Deliv. Rev.* **61** (2), 86–100.

Milad, N. & Morissette, M.C. 2021 Revisiting the role of pulmonary surfactant in chronic inflammatory lung diseases and environmental exposure. *Eur. Respir. Rev.* **30** (162), 210077.

Mitsoulis, E. 2007 Flows of viscoplastic materials: Models and computations. *Rheol. Rev.* **135**, 135–178.

O'Donnell, A.E., Barker, A.F., Ilowite, J.S. & Fick, R.B. 1998 Treatment of idiopathic bronchiectasis with aerosolized recombinant human DNase I. *Chest* **113** (5), 1329–1334.

Ogrosky, H. R. 2021 Linear stability and nonlinear dynamics in a long-wave model of film flows inside a tube in the presence of surfactant. *J. Fluid Mech.* **908**, A23.

Otis, D.R., Johnson, M., Pedley, T.J. & Kamm, R.D. 1990 The effect of surfactant on liquid film stability in peripheral airways. *Adv. Bioeng.* **17**, 55–57.

Otis, D.R., Johnson, M., Pedley, T.J. & Kamm, R.D. 1993 Role of pulmonary surfactant in airway closure: a computational study. *J. Appl. Physiol.* **75** (3), 1323–1333.

Patarin, J., Ghiringhelli, É., Darsy, G., Obamba, M., Bochu, P. & de Saint Vincent, M.R. 2020 Rheological analysis of sputum from patients with chronic bronchial diseases. *Sci. Rep.* **10**, 15865.

Romanò, F., Muradoglu, M. & Grotberg, J. B. 2022 Effect of surfactant in an airway closure model. *Phys. Rev. Fluids* **7**, 093103.

Romanò, F., Muradoglu, M., Fujioka, H. & Grotberg, J.B. 2021 The effect of viscoelasticity in an airway closure model. *J. Fluid Mech.* **913**, A31.

Schürch, S., Bachofen, H. & Possmayer, F. 2001 Surface activity in situ, in vivo, and in the captive bubble surfactometer. *Comp. Biochem. Physiol. Part A Mol. Integr. Physiol.* **129** (1), 195–207.

Shemilt, J.D., Horsley, A., Jensen, O.E., Thompson, A.B. & Whitfield, C.A. 2022 Surface-tension-driven evolution of a viscoplastic liquid coating the interior of a cylindrical tube. *J. Fluid Mech.* **944**, A22.

Stone, H.A. 1990 A simple derivation of the time-dependent convective-diffusion equation for surfactant transport along a deforming interface. *Phys. Fluids A* **2** (1), 111–112.

Tiddens, H.A.W.M., Donaldson, S.H., Rosenfeld, M. & Paré, P.D. 2010 Cystic fibrosis lung disease starts in the small airways: can we treat it more effectively? *Pediatr. Pulmonol.* **45** (2), 107–117.

Walton, I.C. & Bittleston, S.H. 1991 The axial flow of a Bingham plastic in a narrow eccentric annulus. *J. Fluid Mech.* **222**, 39–60.
